# Automated detection of neutralizing SARS-CoV-2 antibodies in minutes using a competitive chemiluminescence immunoassay

**DOI:** 10.1007/s00216-022-04416-6

**Published:** 2022-11-08

**Authors:** Julia Klüpfel, Sandra Paßreiter, Melina Rumpf, Catharina Christa, Hans-Peter Holthoff, Martin Ungerer, Martin Lohse, Percy Knolle, Ulrike Protzer, Martin Elsner, Michael Seidel

**Affiliations:** 1 Institute of Water Chemistry, Chair of Analytical Chemistry and Water Chemistry, Technical University of Munich, Lichtenbergstr. 4, 85748 Garching, Germany; 2 Institute of Virology, Technical University of Munich/Helmholtz Zentrum München, Trogerstr. 30, 81675 Munich, Germany; 3 ISAR Bioscience GmbH, Semmelweisstr. 5, 82152 Planegg, Germany; 4 Institute of Molecular Immunology/Experimental Oncology, Technical University of Munich, Ismaningerstr. 22, 81675 Munich, Germany; 5 German Center for Infection Research (DZIF), 81675 Munich, Germany

**Keywords:** SARS-CoV-2, COVID-19 serology, Protein-receptor interaction, Chemiluminescence immunoassay, Neutralizing antibodies, Competitive immunoassay

## Abstract

The SARS-CoV-2 pandemic has shown the importance of rapid and comprehensive diagnostic tools. While there are numerous rapid antigen tests available, rapid serological assays for the detection of neutralizing antibodies are and will be needed to determine not only the amount of antibodies formed after infection or vaccination but also their neutralizing potential, preventing the cell entry of SARS-CoV-2. Current active-virus neutralization assays require biosafety level 3 facilities, while virus-free surrogate assays are more versatile in applications, but still take typically several hours until results are available. To overcome these disadvantages, we developed a competitive chemiluminescence immunoassay that enables the detection of neutralizing SARS-CoV-2 antibodies within 7 min. The neutralizing antibodies bind to the viral receptor binding domain (RBD) and inhibit the binding to the human angiotensin-converting enzyme 2 (ACE2) receptor. This competitive binding inhibition test was characterized with a set of 80 samples, which could all be classified correctly. The assay results favorably compare to those obtained with a more time-intensive ELISA-based neutralization test and a commercial surrogate neutralization assay. Our test could further be used to detect individuals with a high total IgG antibody titer, but only a low neutralizing titer, as well as for monitoring neutralizing antibodies after vaccinations. This effective performance in SARS-CoV-2 seromonitoring delineates the potential for the test to be adapted to other diseases in the future.

**Graphical Abstract:**

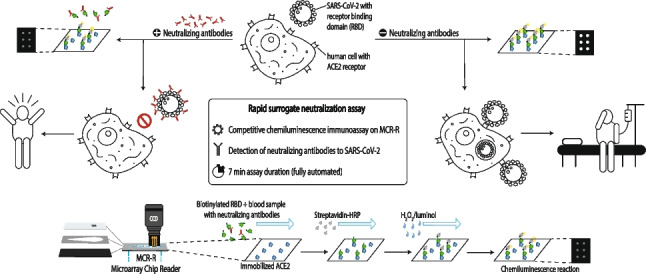

**Supplementary information:**

The online version contains supplementary material available at 10.1007/s00216-022-04416-6.

## Introduction

Since its outbreak in late 2019, the SARS-CoV-2 pandemic affected the lives of billions of people around the world. At the time of writing this manuscript, the World Health Organization (WHO) reported 613 million confirmed cases and 6.5 million deaths [1]. To monitor SARS-CoV-2 infections, especially those that go unnoticed [2], diagnostic methods to determine the presence of antibodies were rapidly developed [3–6]. However, these tests screen for antibodies to multiple epitopes and, therefore, cannot provide information about the effective protective immunity that is gained in the form of those antibodies that truly prevent the cell entry of SARS-CoV-2 [7].

Essential for this cell entry is the binding of the viral receptor binding domain (RBD), located within the SARS-CoV-2 spike protein (S1 fragment), to the human angiotensin-converting enzyme 2 (ACE2) receptor at the cell surface. This receptor is, for example, strongly expressed in lung tissue. Those antibodies that are capable of specifically binding to the RBD and, thereby, of preventing cell entry, are called neutralizing antibodies [8]. Studies have shown that the levels of neutralizing antibodies (nAbs) decrease over time and that certain patient groups even develop only low level of nAbs in the first place [9, 10]. Therefore, the determination of the neutralizing capacity of SARS-CoV-2 antibodies is of great interest for understanding SARS-CoV-2 immunity [11] and for giving recommendations on booster vaccines in point-of-care settings in the future as is currently already done for other infectious diseases.

The standard method for the detection of neutralizing antibodies is plaque reduction neutralization tests (PRNTs) [12]. Here, serum samples are incubated with active virus, and subsequently, eukaryotic cells are infected with the virus and incubated for several days. When evaluating the amount of formed plaques (regions of cell destruction due to viral infection), one can see if neutralization of the virus by antibodies in the serum occurred or if cells were infected at undiminished velocity. This assay principle is not only time-intensive, but also requires highly skilled staff and biosafety level 3 laboratory facilities, which often are not available [12]. An alternative are surrogate assays that do not use active virus but rely on non-infectious viral proteins (especially the spike protein) instead [13]. This makes the assays faster and the use of BSL3 laboratories obsolete, so that assays become accessible for many laboratories. Although these assays might miss some neutralizing antibodies to other proteins than the spike protein and its receptor binding domain, they generally give a good estimate of neutralizing antibodies in a sample compared to virus-based neutralization tests [14–16]. Since 2020, various surrogate assays have been presented in scientific literature or even made commercially available, most of them by applying ELISA techniques [17–20], but also luciferase assays [21] or bead-based Luminex assays [22] can be found. Many of the reported surrogate assays showed a performance equivalent to PRNTs in significantly less time with only a few hours instead of days. Very few examples of rapid, point-of-care neutralization tests with turnaround times below 1 h can be found in terms of lateral flow assays [23, 24] or cellulose pull-down tests [25], but these tests often suffer from bias when readout is done by eye and even digital readout is easily influenced by varying quality of blood samples or the exact time point of readout, sometimes making even relative quantification difficult. But still, such fast assays are required, for example, to test for neutralizing antibodies at a medical practitioner and to then immediately give a booster vaccination if necessary or for verification of vaccination status, border control, or for the screening for possible donors for convalescent plasma [24, 26]. Even though currently, reliable threshold values for reasonable protection are not known yet, this will probably change in the future as it has already been shown that neutralizing antibody levels are highly predictive of immune protection [27]. And already now, the public readily makes use of rapid antibody tests offered by pharmacies which only give information on binding but not on neutralizing antibodies. Here, rapid neutralization assays would be a valuable tool in order to not give people a false feeling of protection in case they have antibodies binding to other motifs on SARS-CoV-2 rather than neutralizing antibodies.

Therefore, we developed a competitive chemiluminescence immunoassay for the measurement of neutralizing SARS-CoV-2 antibodies. Due to the flow-based detection principle and the short sample incubation time, results are available significantly faster than for statically incubated assays.

As a prerequisite for the neutralization assay, first the protein-receptor interaction between SARS-CoV-2 RBD and human ACE2 had to be assessed in detail on the analysis platform Microarray Chip Reader – Research Edition (MCR-R, described in detail in Klüpfel et al. [28]) to be able to define suitable conditions for the subsequent measurement of the inhibition of this binding in a competitive assay. While previous works with this analysis platform included various immunoassay formats for the detection of bacteria [29], small-molecule antibiotics [30, 31], toxins [32, 33], or antibodies [34, 35], for example, by sandwich immunoassay or chip-based amplification [36, 37], no example for the measurement of protein-receptor interaction as well as its inhibition has been presented on the MCR so far. Therefore, this first example of such an assay on the platform opens the door into a broad field with multiple potential applications that have so far been served by other methods including radioligand binding assays, surface plasmon resonance, isothermal titration calorimetry [38], or classical immunoassay techniques like ELISA [39].

**Fig. 1:**
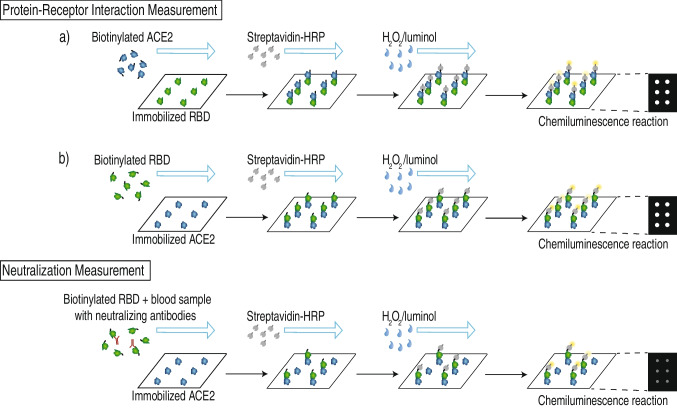
Overview over immunoassay principles for protein-receptor interaction measurements (top) and neutralization antibody measurements (bottom) for SARS-CoV-2 on the MCR-R

Figure 1 shows the measurement principles for the protein-receptor binding assay as well as for the subsequent competitive neutralization assay.

The determination of the protein-receptor interaction is possible in two ways: immobilization of the (a) RBD or (b) ACE2 protein on the chip surface. In a first step, the respective complementary biotinylated protein (ACE2 in the case of (a), RBD in the case of (b)) is injected into the chip, leading to formation of RBD-ACE2 complexes at the chip surface. Subsequently, these complexes can be detected when horseradish peroxidase (HRP)-labeled streptavidin is flushed over the chip surface, because the streptavidin binds to the biotin label and catalyzes a chemiluminescence (CL) reaction in the presence of H_2_O_2_ and luminol resulting in a measurable bright light signal.

Other assay principles and analysis platforms had been used previously for the detection of RBD-ACE2 binding. A similar but more time-consuming approach is interaction measurement by ELISA, which has shown that a sigmoidal binding curve is obtained from the interaction of SARS-CoV-2 RBD and ACE2.

Generally, various examples for the measurement of ligand-receptor interaction using more time-extensive ELISA methods can be found in literature [40–42]. In such bimolecular binding reactions, a hyperbola would be expected when titrating receptor with ligand or vice versa, unless secondary effects influence the binding. A common example for such effects is cooperativity where binding of one ligand molecule to the receptor influences the affinity of subsequent ligand molecules on the receptor [43]. In consequence, a sigmoidal curve is found as has been shown for ELISA measurements of RBD-ACE2 interaction [39, 44].

Thus, these protein-receptor interaction measurements were not only used to find the most suitable orientation of the assay but mainly for the determination of optimal concentrations of the respective protein on the surface and in solution as to give high signal when no neutralizing antibodies are present but to also be susceptible to minimal amounts of inhibition, corresponding to a position at the steepest part of the respective sigmoidal binding curve. As the use of immobilized ACE2 was found to be beneficial regarding necessary reagent concentrations, this orientation was used for inhibition measurements to detect neutralizing antibodies. Additionally, this first assay development stage was also used to evaluate different immunochip materials, showing that amino-modified glass slides were most suitable.

**Fig. 2:**
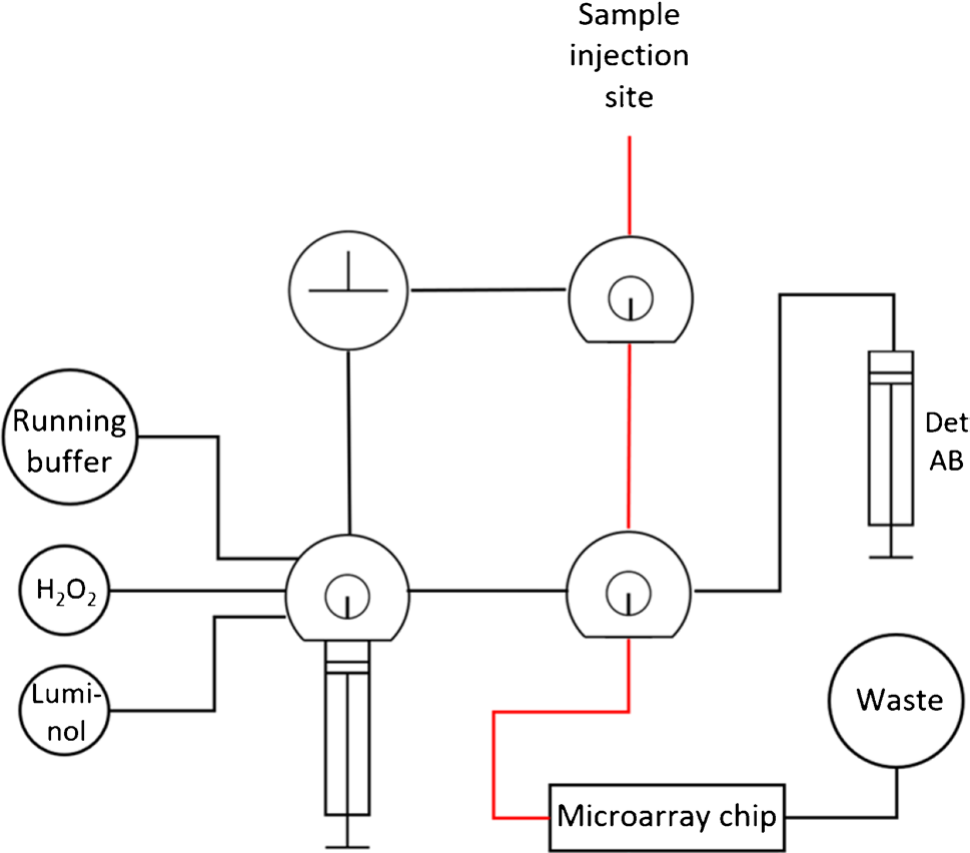
Simplified tubing plan of the MCR-R with newly added sample injection site using a syringe adapter (sample path marked in red); additional tubes not used in the assay are omitted for clarity

After the evaluation of these general conditions, the next development step is the inhibition of the protein-receptor interaction by neutralizing antibodies. For this neutralization assay, a serum sample is mixed with biotinylated RBD and injected into the microarray chip with immobilized ACE2 as shown in Fig. 2 (bottom). As is typical in such competitive assays, the signal will be brighter the fewer neutralizing antibodies are present, because when neutralizing antibodies bind to the biotinylated RBD, they prevent RBD-ACE2 complexes at the chip surface and, therefore inhibit the chemiluminescence signal.

In addition to presenting the first method for detecting protein-receptor interaction and its inhibition with the analysis platform MCR-R, we also present a comparison of different microarray chip materials for their application in these assays. To evaluate the performance of the novel competitive assay for the detection of neutralizing SARS-CoV-2 antibodies, we show the successful measurement of 80 serum samples. We further show that these results also correlate well to a total IgG antibody assay and a neutralization ELISA. Finally, the possibility of monitoring neutralizing antibodies after vaccination is presented. These results show that surrogate neutralization assays can be performed in less than 10 min by competitive chemiluminescence immunoassays using a flow-based detection principle. They open the way to point-of-care diagnostic tests in this field of immune diagnostics.

## Experimental

### Chemicals, reagents, and materials

Standard chemicals were obtained from Sigma-Aldrich, subsidiary of Merck (Darmstadt, Germany), VWR (Rednor, USA) and Carl Roth (Karlsruhe, Germany). Hydrogen peroxide and luminol solution was bought from Cyanagen (Bologna, Italy) in the Elistar Supernova reagent kit. Streptavidin-Peroxidase was purchased from Biozol (VEC-SA-5004). A peroxidase-labeled anti-human IgG antibody (Fc fragment) from goat was obtained from Sigma-Aldrich (A0170, 5.6 mg mL^−1^).

Spotting buffer was produced as described elsewhere [45], while as running buffer, Dulbecco’s phosphate-buffered saline with 0.1% (*v*/*v*) Tween® 20 was used.

### SARS-CoV-2 antigens

Human ACE2 protein was ordered from Sino Biological (Beijing, China). Recombinant SARS-CoV-2 spike RBD proteins with His-tag from wild-type virus (wt RBD) as well as the delta variant (delta RBD) were produced by ISAR Bioscience (Planegg, Germany) with the wt RBD being taken from the S protein nucleotide sequence of the SARS-CoV-2 Wuhan Hu-1 genome (GenBank accession number MN908947, positions 22517 to 23183), while the delta RBD contained the following mutations: L452R and T478K. Details on the procedures were published before [45, 46]. Shortly, CHO cells were transfected with plasmid vectors containing the DNA sequences for the RBD proteins with an added His-tag and subsequently grown at 37 °C. The supernatants were centrifuged and filtered and subsequently purified using HisTrap columns. Protein content after elution was determined by OD_280_ measurement and the relevant fractions were dialyzed.

The biotinylation of wt RBD and human ACE2 was done using the EZ-Link Micro Sulfo-NHS-LC biotinylation kit (Thermo Scientific #21935 or #A39257) with 20-fold molar excess according to the standard procedure instruction, followed by removal of excess biotin by dialysis against 1 L PBS for 16 h at 4 °C, using Slide-A-Lyzer™ Dialysis Cassettes, 7 K MWCO, 0.5 mL (Thermo Scientific #66373).

### Serum and plasma samples

Serum and plasma samples were either purchased from Sigma-Aldrich (Darmstadt, Germany), obtained from the Institute of Molecular Immunology and the Institute of Virology, Technical University of Munich (Munich, Germany) or collected in the course of this study. All procedures were in accordance with the Helsinki Declaration of 1975, as revised in 2000.

All patient data were anonymized before use of the samples. Patient samples were handled in laboratories approved for biosafety level 2.

### Chip surface preparation

The chemiluminescence immunoassays were performed either on glass or polycarbonate (PC) slides or PC foils with surface modifications based on procedures described previously [28, 47, 48], that were applied with some alterations and optimizations. Shortly, glass microscopy slides were surface modified by silanization and subsequent coupling of the polyetheramine Jeffamine® ED-2003. For the optimized production of glass chips, incubation times for acid treatments before silanization were reduced to 15 min, and volumes of silanization reagent and Jeffamine® ED-2003 were reduced to 300 µL to allow for upscaling and lower prices per unit.

PC sheets (1-mm thickness) were prepared using carboxy-modified Jeffamine as detailed in previous works [28, 48] with an alteration of the incubation temperature to 90 °C. Shortly, Jeffamine® ED-2003 was carboxy-modified by coupling of succinic anhydride and subsequently, PC sheets were coated with the molten polymer by screen printing. PC foils (0.25-mm thickness) were treated equally.

### Microarray chip production

Before spotting proteins on the chip surface, most glass chips were activated using *N,N*′-disuccinimidyl carbonate (DSC) activation [45]. A mixture of 8 mg N*,N*′-disuccinimidyl carbonate, 0.4 mg 4-(dimethylamino)pyridine and 12.5 µL triethylamine in 160 µL dimethylformamide per chip was prepared, and 300 µL of this mixture were incubated between two functionalized glass slides in sandwich principle at RT for 30 min, followed by manual cleaning and sonication in methanol. After drying, they were either directly used for spotting or stored at 4 °C until spotting.

Spotting solutions were prepared as antigens or positive control antibody diluted with spotting buffer as described earlier [45]. As positive control, anti-peroxidase antibody was used, while as negative control spotting buffer was applied.

For spotting without previous activation of the chip surface (EDC/s-NHS spotting [28], applied to all PC chips and certain glass chips), 1 mg mL^−1^ 1-ethyl-3-(3-dimethylaminopropyl)carbodiimide (EDC) and 1 mg mL^−1^ N-hydroxysulfosuccinimide (s-NHS) were added to spotting buffer and mixed with prediluted or undiluted antigen and positive control solutions (50% v/v).

Spotting was then done using the the BioOdyssey Calligrapher miniarrayer from Bio-Rad (Hercules, USA) with a SNS9 spotting pin using the same procedure applied for our previous SARS-CoV-2 assays [28]. In short, five replicates per spot were transferred onto the glass or PC chips (transverse to intended flow direction on the microarray chip) with up to 20 different solutions in flow direction (spotting rows). The chips were assembled with a polyoxymethylene (POM) carrier containing in- and outlet holes and a double-sided adhesive foil with a cut-out flow channel and stored at 4 °C until measurement.

### Microarray measurements for total IgG antibody detection

Microarray measurements were done on the microarray platform MCR-R, which was obtained from GWK Präzisionstechnik (Munich, Germany) and has been described in detail in a previous publication [28]. As presented there, the device had to be flushed at the beginning of a measurement day and was subsequently loaded with the necessary reagents, followed by a darkframe image to correct for camera background. For measurements, plasma or serum samples were prepared by diluting 20 µL of sample with 205 µL PBST and a measurement chip was inserted into the chip unit. The measurement was started, and the sample was injected into a valve of the MCR-R using an adapter for low residual volume syringes. The sample filled the tubing from valve to chip and was then pushed over the chip by a syringe pump transporting running buffer. The overall simplified tubing plan is shown in Fig. 2, while detailed information on the measurement program is summarized in the Supporting Material (Section S1).

To obtain an optimal interaction between sample and immobilized antigens, a stopped flow was applied, allowing for the incubation of small sample aliquots on the chip for 5 s. After the sample transport, the chip was flushed slowly with peroxidase-labeled anti-human IgG antibody, followed by chemiluminescence reagents. The camera exposure time was 60 s; afterwards the tubing as well as the sample injection adapter were flushed thoroughly, giving a total time of 6.5 min per measurement including manual steps.

### Microarray measurements for protein–protein interaction measurement

To detect protein–protein interaction between human ACE2 and RBD, a measurement program with a duration of 3 min 45 s that had previously been used for an antibody assay, was applied [28]. 40 µL samples of either biotinylated RBD or ACE2 were prepared in concentrations of 0, 0.05, 0.1, 0.5, 1, 5, 10, 20 µg mL^−1^ or 0, 0.5, 1, 5, 10, and 50 µg mL^−1^, respectively. For measurements, a sample was injected into a chip directly, the chip was inserted into the MCR-R, and the measurement was started immediately.

### Microarray measurements for neutralizing antibody detection

To detect the inhibition of RBD-ACE2 binding by neutralizing antibodies, a solution of biotinylated RBD in PBST with a concentration of 10 µg mL^−1^ was prepared. 20 µL of this solution were mixed with 20 µL of serum, plasma, or whole blood sample and injected into a microarray chip that was then inserted into the MCR-R, where the automated measurement was started immediately. The measurement program was equal to the one used for protein–protein interaction measurements.

### Neutralization ELISA measurements

The neutralization ELISA was conducted as described previously by Richardson et al. [46]. Shortly, ELISA plates were coated with 60 ng ACE2 per well for 1 h, followed by washing (with PBST) and blocking steps (milk powder solution for 1 h). Serum samples were incubated in 1:2 dilution together with 18 ng of biotinylated RBD per well for 1 h, followed by incubation of streptavidin peroxidase for 1 h. After addition of TMB substrate and stopping of the reaction with H_2_SO_4_, absorbance was determined at a wavelength of 450 nm.

### Surrogate neutralization assay on YHLO iFlash 1800

A commercial and certified surrogate paramagnetic particle chemiluminescence immunoassay (CLIA) by the manufacturer YHLO Biotechnology (Shenzhen, China) for quantification of neutralizing antibodies was performed. Neutralizing antibodies in sera are linked to SARS-CoV-2 RBD antigen-coated paramagnetic microparticles. The remaining microparticles are competitively bound by acridinium-ester labeled ACE2 conjugates. The number of neutralizing antibodies is calculated in AU mL^−1^ [arbitrary units per milliliter] and correlates inversely to the reaction mixtures relative light units (RLU) [49, 50]. The lower limit of quantification is 4 AU mL^−1^, and the upper limit of quantification is 800 AU mL^−1^. Seropositivity is given for values above 10 AU mL^−1^ according to the manufacturer’s instructions. Results can be adapted to WHO International Standard (NIBSC code 20/136) by conversion (AU mL^−1^ × 2.4 = IU mL^−1^ [international units per milliliter]).

### Data evaluation

The detected CL signals were corrected with the previously recorded blank image, stored as txt-files, and processed with the evaluation software “MCR-Analyser” (Martin Knopp, Munich, Germany) [51]. On the background-corrected CL images, a grid was set to define the position of the spots. For each spot, the mean value of the ten brightest pixels was calculated. Means and standard deviations were calculated for the five replicates per row and spots that deviated more than 10% from the mean were excluded.

The resulting mean values and standard deviations for all rows were used for further analysis and graphical evaluation using Python 3.

## Results and discussion

### Measurement of SARS-CoV-2–ACE2 interaction

To pre-define obligatory parameters for the neutralization assay, first a protein-receptor interaction assay was established, aimed at finding optimal concentrations of the used proteins as well as optimal chip material.

**Fig. 3:**
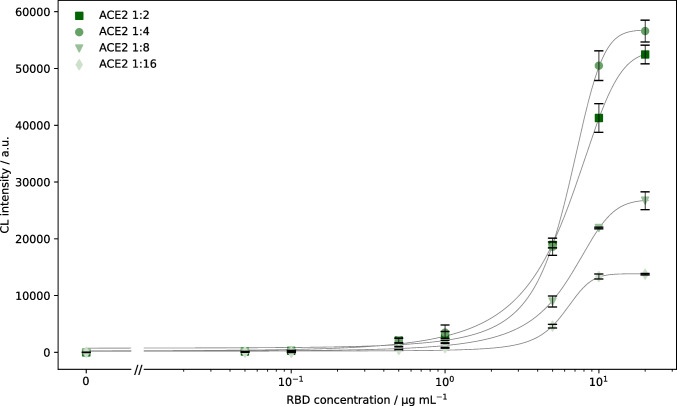
Comparison of different immobilized ACE2 concentrations for determination of ACE2-RBD binding (CL signals are background*-*corrected, error bars show standard deviations of triplicate measurements, curves were fitted using 4-parameter logistic fit, linear axis scaling is used left of the axis break to include 0 µg mL^−^.^1^)

First, it was tested whether the immobilization of ACE2 or RBD was more suitable and what dilution of the immobilized protein was optimal. For immobilized ACE2, twofold dilutions between 1:2 (0.5 mg mL^−1^) and 1:16 were spotted on the same chips and different concentrations of biotinylated RBD ranging from 0 to 20 µg mL^−1^ were added in an automated non-competitive immunoassay, giving the CL intensity curves shown in Fig. 3. While the 1:2 and 1:4 dilution gave comparable results, the higher dilutions gave significantly lower signals. In sigmoidal interaction curves, optimally the increasing part of the curve should span over a concentration range of at least one log value, while at the same time also covering a big range of intensity values. These criteria are best met by the highest tested concentrations. As the 1:2 and 1:4 dilution gave comparable results, it is assumed that a maximum occupation of the small spot surface was obtained with the 1:4 dilution, and additional ACE2 could not be bound covalently to the surface. Still, a 1:2 dilution of ACE2 was used for further experiments to make sure that the maximum possible amount of protein was bound to the surface. The respective EC50 value for the 1:2 dilution was 5.7 µg mL^−1^, which was considered a suitable concentration of RBD for the use in the inhibition measurements of the subsequent neutralization assay.

In contrast to immobilizing ACE2, it is also generally possible to immobilize the SARS-CoV-2 RBD. While we were able to prove the general applicability of this principle (results shown in Supplementary Material) and also its advantages for future simultaneous evaluation of neutralizing antibody responses to different SARS-CoV-2 variants, we focused on the more economic and currently also more reliable principle with immobilized ACE2.

Apart from the assay orientation and reagent concentrations, also different chip materials, namely amino-modified glass and carboxy-modified polycarbonate in different thicknesses (1 mm and 0.25 mm), were evaluated. Similar experiments had been done for a total IgG antibody assay for SARS-CoV-2 before (results shown in Supporting Material), showing that glass chips performed best, followed by thin PC foils. The re-assessment was done as the detection mechanism for the neutralization assay differs from that of the total IgG antibody assay; thus, a different outcome would be possible.

Therefore, human ACE2 (0.5 mg mL^−1^) was immobilized on DSC glass chips as well as on PC sheet (1 mm) and foil (0.25 mm) chips and tested with biotinylated RBD as described before. To additionally account for the influence of interaction time between RBD and ACE2, an incubation of RBD in the chip for 2 min before starting the measurement was tested, giving a notably higher interaction time compared to the standard interaction time of 10 s.

**Fig. 4:**
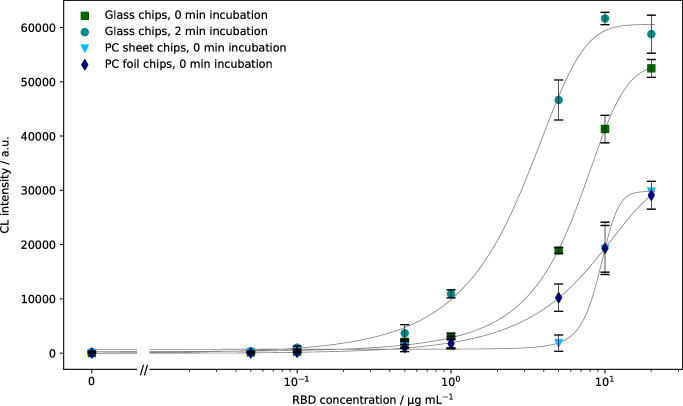
Comparison of different chip materials and incubation times for determination of ACE2-RBD binding (CL signals are background-corrected, error bars show standard deviations of triplicate measurements, curves were fitted using 4-parameter logistic fit, linear axis scaling is used left of the axis break to include 0 µg mL^*−*^.^1^)

The resulting CL values are shown in Fig. 4, indicating that for the glass chips higher endpoint CL values were obtained (about 60,000 a.u. compared to 30,000 a.u.). Especially for the PC sheet chips a very steep curve was found, indicating a very small working range for the subsequent neutralization assay. For PC foil chips, no endpoint plateau was reached, indicating that even higher concentrations of RBD would be necessary. In consequence, glass chips again were considered more suitable than PC chips for both interaction and the following inhibition measurements. The reason for this outcome can be found in the different immobilization strategy on PC, possibly resulting in insufficient amounts or inept orientation of immobilized ACE2.

For the tested incubation times on glass chips, it was found that after a 2-min incubation, the concentration of streptavidin-peroxidase could be significantly lowered (1:10,000 instead of 1:2500 dilution) while still giving comparable endpoint CL intensities as the 10-s incubation. Furthermore, the longer incubation led to a significantly reduced EC50 value of 1.8 µg mL^−1^ compared to 5.7 µg mL^−1^. Therefore, prolonged incubation is a helpful tool if one wants to evaluate solely a protein-receptor interaction, but inhibition experiments showed that the 2-min incubation was not suitable for the detection of neutralizing antibodies as the inhibition was overestimated, even in negative samples, possibly due to non-specific binding of serum proteins to either immobilized ACE2 or biotinylated RBD. Hence, glass chips with 10-s sample incubation were used for further evaluations.

### Concentration dependency of neutralization measurements

As a next development step, it was tested what influence the addition of SARS-CoV-2 seropositive and negative samples had on the obtained signal and whether the signal inhibition after addition of positive samples was concentration-dependent. Therefore, a competitive binding inhibition assay format was used. Chips were spotted with ACE2 (0.5 mg mL^−1^) and biotinylated RBD was mixed with either a positive sample in different dilutions or a negative sample to a final RBD concentration of 5 mg mL^−1^ to be close to the previously defined EC50 value. It was expected that with a SARS-CoV-2 seronegative sample no signal change would occur, while for a positive sample, the signal would decline in a concentration-dependent way in comparison to a measurement without serum.

**Fig. 5:**
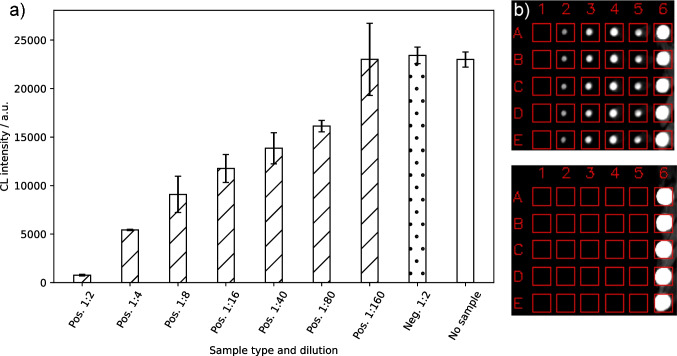
**a** Inhibition measurements using different dilutions of a SARS-CoV-2 seropositive sample, a negative control sample and a measurement without addition of serum sample (CL signals are background-corrected, error bars show standard deviations of triplicate measurements), **b** measurement images for a seronegative (top) and seropositive (bottom) sample at 1:2 dilution (Column 1: negative control, 2–5: ACE2 in different dilutions from 1:16 to 1:2, 6: positive control)

Figure 5 shows the measurement results that confirmed these expectations. While a 1:2 diluted negative sample gave results around 25,000 a.u.—just like a measurement with 5 µg mL^−1^ RBD without serum addition—the positive sample gave a significantly reduced signal in a 1:2 dilution (below 1,000 a.u.) and showed a concentration-dependent signal increase upon higher dilutions until a 1:160 dilution could not be distinguished from a negative sample anymore.

This experiment showed that the measurement of neutralizing antibodies is generally possible with the presented assay principle and that positive samples can be detected over a relatively broad concentration range, while negative samples give high intensities as anticipated. In continuation, the assay was tested with a higher number of positive and negative samples to reveal its applicability as some positive samples might only lead to a very slight signal reduction while negative samples could possibly inhibit due to non-specific binding to either RBD or ACE2.

### Detection of neutralizing antibodies in blood samples and comparison with alternative methods

**Fig. 6:**
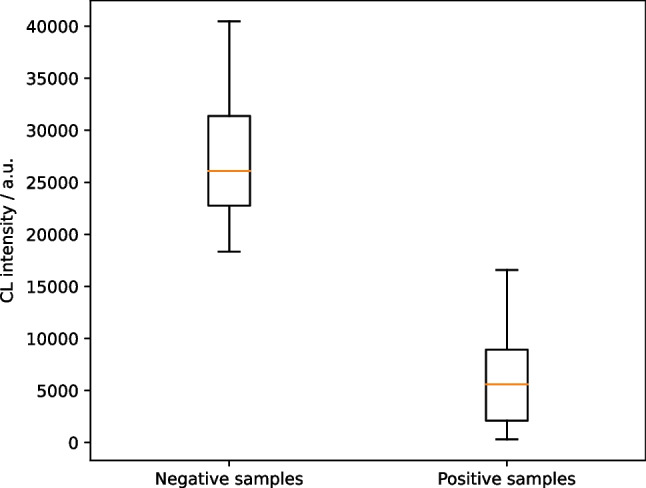
Neutralization measurements of 33 SARS-CoV-2 seronegative and 47 positive samples (CL signals are background-corrected)

To evaluate the performance of the neutralization assay, a total of 80 samples (33 SARS-CoV-2 seronegative, 47 positive) were measured. They were well distinguishable with positive samples giving low CL intensities and negative ones giving high intensities as shown in Fig. 6. A two-tailed unpaired *t* test on the data resulted in a *P* value < 0.0001 for a significance level of *α* = 0.05, emphasizing the statistically significant differences between the sample groups.

**Fig. 7:**
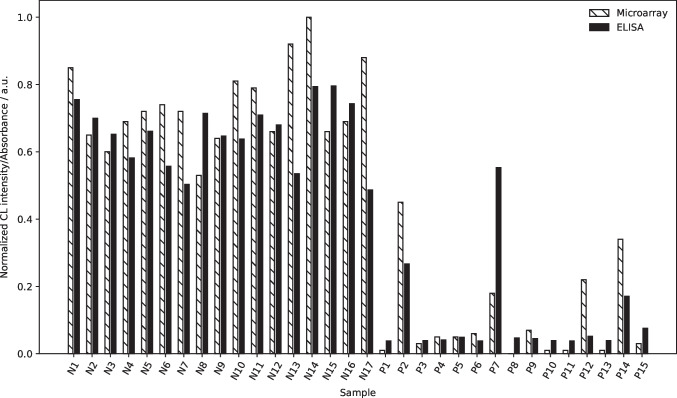
Comparison of neutralization ELISA and neutralization CL-MIA (CL signals are background-corrected and normalized for easier comparison, ELISA results are given as measured absorbance)

In parallel, for 15 positive and 17 negative samples, comparison measurements were done for confirmation using a surrogate neutralization ELISA. While the measured signals differed for some samples (see Fig. 7), the general trends are very similar in both assays. The differences for some samples can be explained with the different interaction times: while in the statically incubated ELISA assay the sample is let to interact for 1 h, in our neutralization chemiluminescence immunoassay, the interaction time is only a few seconds until the chip is flushed and the detection process is started. Nonetheless, the accordance between the two neutralization assays is very high despite the different assay principles.

To investigate the correlation between the total amount of IgG antibodies and the neutralizing activity of antibodies, 57 samples were tested with a total IgG antibody CL-MIA detecting all antibodies binding to SARS-CoV-2 RBD but not necessarily inhibiting the binding of RBD to ACE2. This assay type had been developed on the MCR-R before [45] and was now improved by using a novel injection approach as detailed in the experimental section to allow for higher reproducibility and lower background intensities. With this procedure, the influence of non-specific binding could be reduced significantly, which had been a problem in some samples before as human IgG binds to surfaces readily and will be detected in the total IgG antibody assay with the anti-IgG detection antibody leading to the possibility of false positive results. For the neutralization assay, in contrast, the direct chip injection procedure could be sustained as not IgG but biotinylated RBD is detected.

**Fig. 8:**
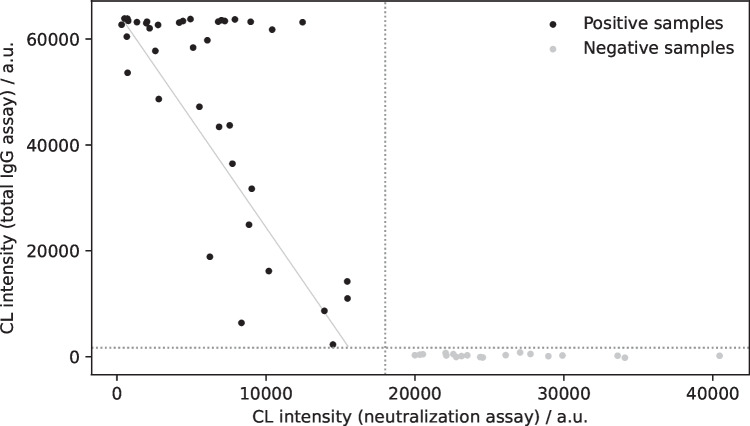
Correlation of results from total IgG antibody assay and neutralization assay (CL signals are background-corrected, dotted lines show separation between positive and negative samples, solid line shows correlation between total antibody signal and neutralization signal in positive samples)

Figure 8 shows the correlation between the neutralization assay and total IgG antibody assay signal. Positive and negative samples can be separated clearly. All negative samples give high signals in the neutralization assay and low signal in the total IgG antibody assay. For many positive samples, also a correlation between both assays is visible (indicated by the gray diagonal line in Fig. 8), but for some samples, very high intensities (approx. 65,000 a.u.) were measured in the total IgG antibody assay while only giving intermediate signal in the range of 5,000–15,000 a.u. in the neutralization assay while a very low signal of approx. 1,000 a.u. would have been expected. This indicates that there were antibodies in that samples that could bind to RBD but not in an epitope that would inhibit the binding of RBD to ACE2. This shows the importance of neutralization tests as total IgG antibody tests do not necessarily give an indication on the protective effect of these antibodies, whereas neutralization assays have proven to give important additional information.

To find out whether the assay could be carried out in a point-of-care manner to gain important information fast and without the need of a specialized laboratory, measurements in whole blood were done. For both positive and negative samples, comparable results were found in whole blood and plasma when considering the hematocrit value and therefore diluting the plasma sample stronger than the whole blood. Therefore, point-of-care applications of the neutralization immunoassay by performing measurements on a drop of capillary blood appear to be a viable option in the future.

Hence, we could show that the measured CL signals are concentration-dependent, and results are well comparable with an alternative surrogate assay format and relatable to total IgG measurements, making the assay very promising for a true quantitative application using international reference standards. While calibration of the assay with such standard material would be easily possible, the respective WHO standard (NIBSC code 20/136 [52], issued in 2020) was not available due to depletion of stocks during the time of our measurements, while a replacement had not yet been issued [53]. Therefore, we compared our results to a commercial surrogate neutralization assay from YHLO, allowing for quantification of neutralizing antibodies in the range of 4 to 800 AU mL^−1^. A total of 64 samples were analyzed using both assays, showing a good correlation between both assays. Among 31 samples that had been pre-classified as seronegative and were also found as negative in the neutralization assay on the MCR-R, one sample taken from a person 14 days after the first SARS-CoV-2 vaccination dose was found to contain neutralizing antibodies by the YHLO assay. This discrepancy can be credited to a slightly higher sensitivity of the YHLO assay due to its longer incubation time, while our neutralization assay cannot detect extremely low antibody numbers shortly after the onset of antibody production. Additionally, YHLO and MCR measurements were done on different sample aliquots that were exposed to different storage conditions, possibly influencing the sample quality. The remaining 30 negative samples were concludingly found as negative in both neutralization assays. For the 33 tested seropositive samples, concentrations between 26 and 800 AU mL^−1^ (upper limit of quantification) were obtained in the YHLO assay with a good correlation to the results obtained on the MCR-R. Correlation analysis resulted in a Pearson R of − 0.87 (negative correlation between AU mL^−1^ and chemiluminescence signal was expected due to the competitive assay format with decreasing signal for increasing antibody concentration). A graphical representation of the correlation can be found in the Supplementary Material (Fig. S4). The CL values obtained for samples at or above the upper limit of quantification for the YHLO assay range over a relatively large range down to CL values of few 100 a.u., indicating that the linear range of the novel assay on the MCR-R might be shifted to even higher neutralizing antibody titers, making it powerful for the analysis of strongly positive samples as are expected shortly after vaccination, while already being able to correctly identify samples with significantly lower titers.

### Monitoring of neutralizing antibodies after vaccination

As the general detection of neutralizing antibodies was shown to be possible with our assay, we tested if it was applicable in the monitoring of neutralizing antibodies after vaccination. It is well known that antibody titers drop over time, so it was tested whether this drop could also be observed with our neutralization assay. Therefore, a total of 11 samples from the same person were measured—the first one was taken 7 days before the first Pfizer/Biontech vaccine dose, followed by a sample 7 days after the first and second dose. The first two doses were administered 21 days apart, while the third dose was given 237 days after the first. The first and the last tested sample were taken 454 days apart.

**Fig. 9:**
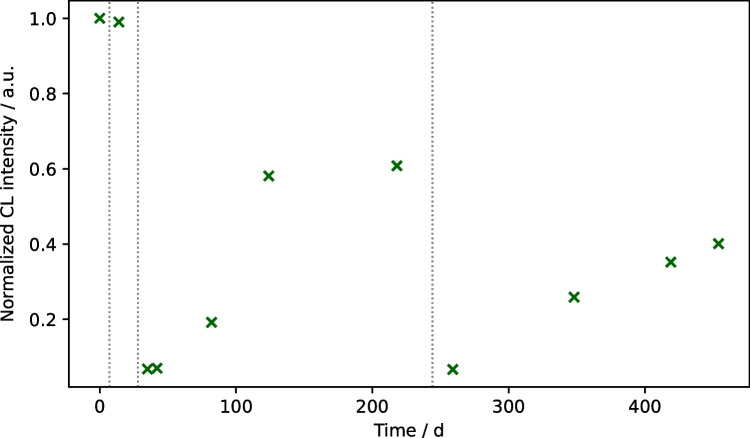
Neutralizing antibody monitoring after SARS-CoV-2 vaccinations; vertical lines indicate vaccine doses (CL signals are background-corrected and normalized with respect to the maximum signal)

The results for the neutralization measurements are shown in Fig. 9. For the first two samples, very high CL intensities are obtained, as no antibodies had been formed yet, while for the third sample, the intensity was as low as 0.07, showing that after the second vaccine dose, a high amount of neutralizing antibodies had been formed. Over the following weeks, the intensity increases again as the antibody titer drops, reaching a value of 0.61 211 days after the first vaccination. A significant increase in neutralizing antibodies was again seen at the measurement date 15 days after the third vaccination with a value of 0.06. In the following, the measured intensities increased again, but at a lower rate compared to the increase after two doses of vaccine so that still a value of 0.40 was found 210 days after the third vaccination. This is an indication of improved sustainability of neutralizing antibodies formed after booster vaccination.

Therefore, it was shown that a monitoring of neutralizing antibodies after vaccinations is possible with our assay and that also changes in the rate of antibody decline after subsequent booster vaccinations can be monitored.

## Conclusion

We were able to develop a rapid method for the detection of neutralizing antibodies in blood samples. While we currently only applied the method to detect SARS-CoV-2 immunity, it can be easily adapted to various other diseases in the future. To further improve the assay for SARS-CoV-2, future research will focus on the use of immobilized RBD to allow for a simple determination of EC50 values and binding behavior for different variants as well as on the applicability of different chip materials. While so far PC chips were not competitive compared to glass chips, it was successfully shown that they could generally be used so that surface optimization might be fruitful.

More importantly, to our present knowledge, the neutralization assay on the MCR-R is faster than all other published surrogate neutralization assays with its measurement duration of 7 min. To stress the general relevance of neutralization assays, we showed that the determination of neutralizing antibody titers is crucial to detect the true protective effect of antibodies as not all persons with a high total antibody count also showed high neutralizing titers, whereas only neutralizing antibodies can help to prevent the infection of cells with SARS-CoV-2.

The neutralization CL-MIA showed outstanding performance in the measurement of serum samples from 80 persons and was proven to give results comparable to a neutralization ELISA and a commercial chemiluminescence immunoassay for neutralization measurements, while being significantly faster. It can thus also be applied to various questions like the monitoring of neutralizing antibodies after vaccinations or infections and can also be used in a point-of-care manner due to its simplicity and speed. In the future, also a quantitative evaluation of measurements will be possible as soon as a new international reference standard will be available. Therefore, it might be applied in pharmacies or at medical practices to give information about the presence of neutralizing antibodies and the need for booster vaccinations on-site.

We here present a powerful tool for the detection of neutralizing antibodies to SARS-CoV-2 that has a huge potential for future applications with respect to other diseases.

## Supplementary information

Below is the link to the electronic supplementary material.

Supplementary file1 (PDF 323 KB)

## Data Availability

Data will be made available upon reasonable request.
