# Supplementary material for: Automated detection of neutralizing SARS-CoV-2 antibodies in minutes using a competitive chemiluminescence immunoassay
Source: Anal Bioanal Chem. 2022 Nov 8;415(3):391–404. doi: 10.1007/s00216-022-04416-6 (PMC9643999; doi:10.1007/s00216-022-04416-6)
Supplement: Supplementary file 1 — Supplementary file1 (PDF 323 KB) [file 216_2022_4416_MOESM1_ESM.pdf]

Automated detection of neutralizing SARS-CoV-2 antibodies in minutes using a competitive chemiluminescence immunoassay

Julia Klüpfel[1‡], Sandra Paßreiter[1‡], Melina Rumpf[1], Catharina Christa[2], Hans-Peter Holthoff[3], Martin Ungerer[3], Martin Lohse[3], Percy Knolle[4], Ulrike Protzer[2,5], Martin Elsner[1], Michael Seidel[1]*

[1]Institute of Water Chemistry, Chair of Analytical Chemistry and Water Chemistry, Technical University of Munich, Lichtenbergstr. 4, 85748 Garching, Germany

[2]Institute of Virology, Technical University of Munich / Helmholtz Zentrum München, Trogerstr. 30, 81675 München

[3]ISAR Bioscience GmbH, Semmelweisstr. 5, 82152 Planegg

[4]Institute of Molecular Immunology/ Experimental Oncology, Technical University of Munich, Ismaningerstr. 22, 81675 München

[5]German Center for Infection Research (DZIF), 81675 München

‡ These authors contributed equally.

***Corresponding author: michael.seidel@mytum.de, tel: +49-89-289-54506**

Content:

S1. Supplemental information on the assay steps for the stopped-flow assay for total IgG measurement on the MCR-R

S2. Supplemental data on the comparison of different microarray chip materials and protein immobilization strategies

S3. Supplemental data for ACE2-RBD interaction measurements with immobilized RBD

S4. Comparison measurements with commercial surrogate neutralization assay

S1. Stopped-flow assay for total IgG measurement on the MCR-R

The total IgG antibody test was carried out in a stopped-flow manner, details on volumes and flow velocities can be found in Table S1. After injection of the sample into the device, the sample was pushed over the chip slowly in increments of 80 µL. After each increment, the flow was stopped for 5 s to allow for the sample to interact with the immobilized proteins. A total volume of 400 µL was transported over the chip consisting of 225 µL sample followed by an additional 175 µL to flush sample residues that might have resided in the tubing over the chip. Subsequently, flushing of the chip with running buffer was done, followed by the slow transport of the detection antibody, peroxidase-labelled anti human IgG antibody, over the chip. After another flushing step, the CL reaction was started by filling the chip with hydrogen peroxide and luminol with an immediate image recording by the CCD camera, followed by final flushing steps.

Table S1 Main assay steps for total anti-SARS-CoV-2 IgG assay on the MCR-R with details of used volumes and flow rates.

Step	Volume	Flow rate	Comment
Flushing of chip	2500 µL	500 µL s^{-1}	
Manual sample injection	225 µL	-	Manually via adapter
Sample transport	400 µL<br>(5 increments, 5 s pause)	10 µL s^{-1}	
Flushing	200 µL<br>2000 µL	10 µL s^{-1}<br>500 µL s^{-1}	
Injection of HRP-labelled anti IgG antibody	115 µL<br>800 µL	50 µL s^{-1}<br>10 µL s^{-1}	
Flushing	2000 µL	500 µL s^{-1}	
CL reagents injection	400 µL	150 µL s^{-1}	
Image acquisition	-	-	60 s exposure
Cleaning of sample input	2000 µL	-	Manually via adapter
Flushing of whole system	7500 µL	250 µL s^{-1}	

S2. Comparison of different chip materials and immobilization principles for use on the MCR-R

Previously, the MCR series had been used with polycarbonate and glass microarray chips for various applications [1–5] but no direct comparison of different chip types in one assay on the same platform had been done before. Therefore, we tested three different materials and two different activation strategies, namely glass chips, PC sheet chips with a thickness of 1 mm, and PC foil chips with a thickness of 0.25 mm. Glass chips were activated with DSC (whole surface activation) as well as with EDC/s-NHS (spot activation only), while for PC only EDC/s-NHS activation was feasible as PC does not tolerate the solvents necessary for DSC activation. Details on the experimental implementation can be found in the Materials and Methods section. The differences between the activation strategies on the molecular level are depicted in Figure S1.

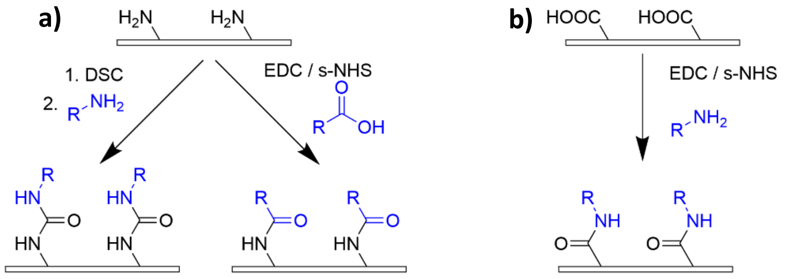

Figure S1 Activation strategies used for **a)** glass and **b)** PC microarray chips.

All chip types were then used for measurements of samples from SARS-CoV-2 naive, vaccinated and reconvalescent persons as shown in Figure S2. The measurements were done on the MCR-R as described previously [6].

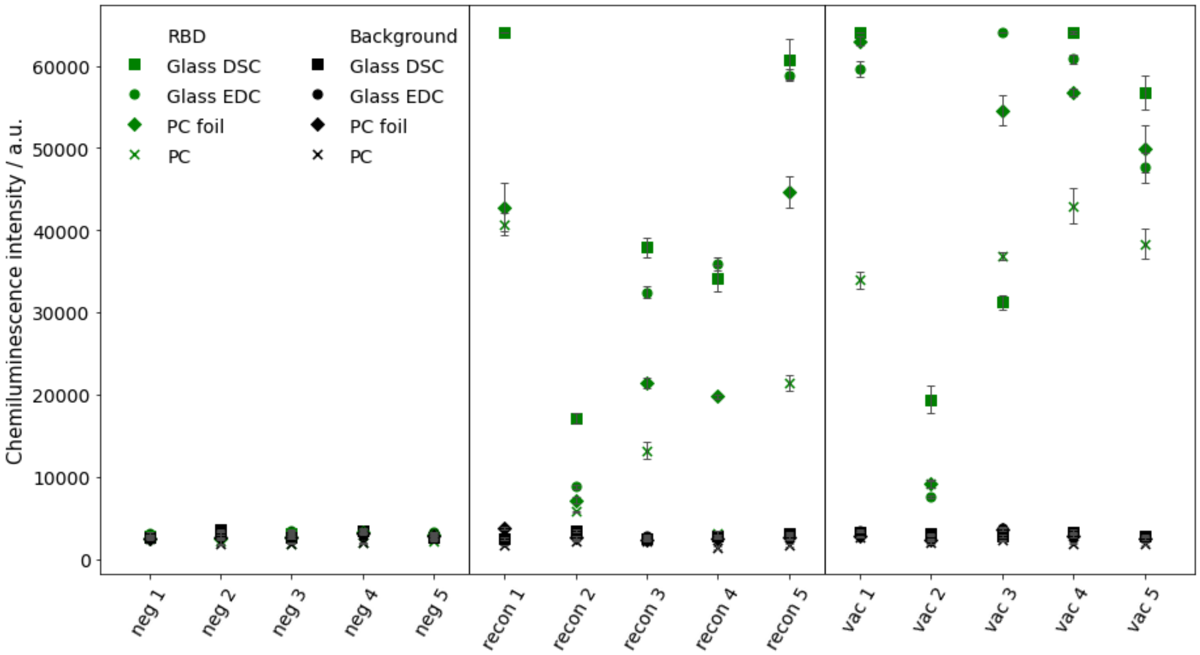

Figure S2 Comparison of measurement results for total antibody detection to SARS-CoV-2 RBD with positive (vaccinated/reconvalescent) and negative samples. RBD is immobilized on glass carrier, PC foils, and PC sheets (error bars show standard deviations of spot replicates on one chip, n = 5).

While the background values as well as the results for negative samples are comparable on all tested chip types, it is clearly visible that for most of the positive samples the highest intensities were measured on glass chips, followed by the two types of polycarbonate chips. Regarding the different activation methods on glass, the DSC activation overall gave slightly higher values compared to the EDC/s-NHS activation with very few exceptions as for example in sample vac 3, which can be attributed to a defect in this single chip. Therefore, DSC activation is considered the optimal activation method for protein immobilization on glass chips as here the protein itself is not compromised by possible crosslinking during the spotting process in contrast to the EDC/s-NHS strategy.

In PC chips, the EDC/s-NHS is used to activate the carboxy groups on the chip surface and let it react with amine groups on the proteins. This procedure generally led to lower intensities compared to glass chips, possibly due to a lower density of functional groups on the surface as during the carboxy coupling of Jeffamine double-substituted polymer that cannot be bound to the surface might result. Additionally, the screen-printing process might be less efficient than the melting process used for the coating of glass chips. Nevertheless, the PC foil chips often came close to the glass chip signals, while the thicker PC sheet chips generally showed a lower performance. A possible reason is the more even surface of foil chips after coating due to the better heat distribution during the incubation after screen printing.

As was detailed in the experimental section and previous publications [6], PC chips have significant advantages with regard to work expenditure and production time. On the other hand, an advantage of DSC-activated glass chips is the possibility to store activated chips at 4 °C for several weeks, while the spotting solutions can also be used for prolonged time as no activating reagents are added. In contrast, in EDC/s-NHS activation, spotting must be done within a few hours after preparation of the spotting solutions to prevent activity loss of the s-NHS ester intermediate. Performance-wise, glass

chips showed superior performance for the total SARS-CoV-2 antibody assay as shown here. But as especially PC foil chips showed relatively comparable results for some samples, chip material evaluations should be repeated whenever new immunoassays are developed.

S3. ACE2-RBD interaction measurements with immobilized RBD

While generally, we used immobilized ACE2 for interaction measurements, the immobilization of RBD is also possible. This principle would have the advantage of immobilizing different RBD variants on the same chip giving additional information within one measurement. To test the behavior of different variants in interaction measurements, wildtype RBD as well as delta RBD were immobilized in tenfold dilutions over a broad concentration range from 800 µg mL^{1} (1:2 dilution) to 8 µg mL^{-1} (1:200 dilution). This wide concentration range was chosen to make differences between the different mutants more apparent. Different concentrations of biotinylated ACE2 were added in a range from 0 to 50 µg mL^{-1}, again resulting in sigmoidal binding curves as shown in Figure 4. For the highest dilution (1:200) only a relatively low signal is obtained at the highest tested ACE2 concentrations, but for 1:20 and 1:2 dilutions stronger signals are obtained already at lower ACE2 concentrations. Especially for the 1:20 dilutions a notable difference between wt RBD and delta RBD is noticeable with delta giving significantly higher signals and at the same time lower EC50 (12.1 µg mL^{-1} compared to 23.9 µg mL^{-1}), possibly indicating a higher affinity of delta RBD to ACE2 compared to wt RBD as one would expect and as has been shown in affinity studies before [7, 8]. Still, more thorough studies on the binding of different RBD variants to ACE2 will be done on the MCR-R in the future to complement these preliminary findings.

Despite these promising results with immobilized RBD, it was decided to conduct the neutralization measurements with immobilized ACE2 and a biotinylated RBD concentration of 5 µg/mL as that assay format showed significantly lower EC50 values, while the relatively high amounts of biotinylated ACE2 that would have been necessary for the alternate assay were considered at the present status of the assay development too cost intensive and, additionally, the measurement of neutralizing antibodies was less reliable (results not shown). This difference might be attributed to sterical reasons, and it may be a consequence of the different principle of the neutralization assay that is not based on RBD-antibody binding in the liquid phase and thus reduction of the available RBD amount but rather on competition on the chip surface. The optimization of this assay towards lower ACE2 amounts and its use in neutralization measurements will be objective of future research.

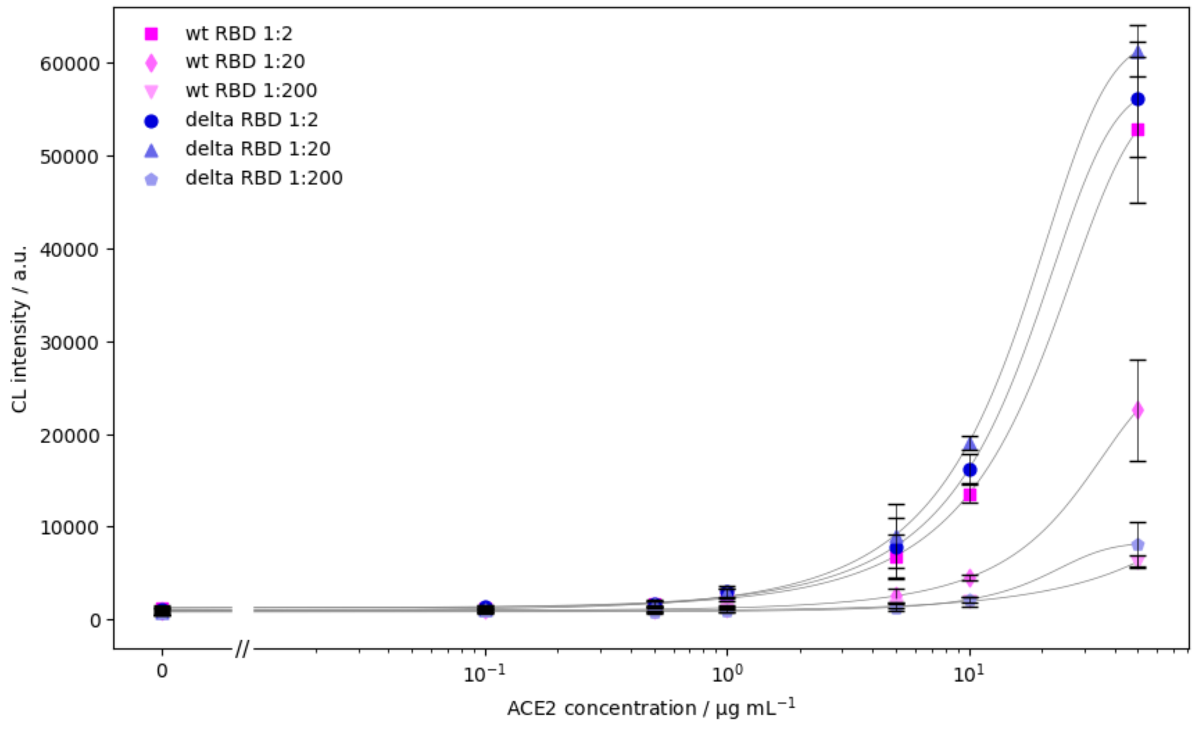

Figure S3 Comparison of different immobilized RBD mutants and concentrations for determination of ACE2-RBD binding (CL signals are background-corrected, error bars show standard deviations of triplicate measurements, curves were fitted using 4-parameter logistic fit, linear axis scaling is used left of the axis break to include 0 µg mL^{-1}).

S4. Comparison measurements with commercial surrogate neutralization assay

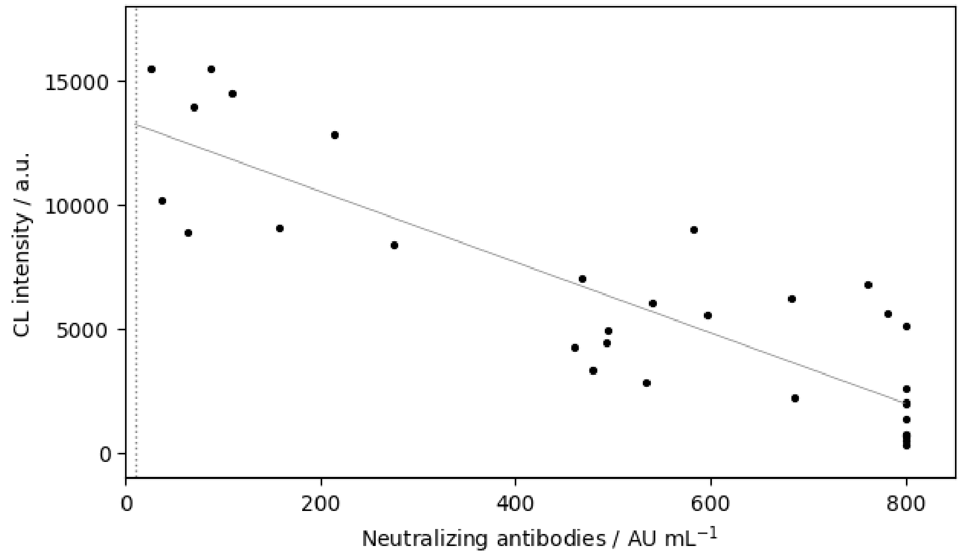

Figure S4 Correlation between neutralizing antibody measurements of 33 seropositive samples in AU mL^{-1} (measured by YHLO iFlash neutralization assay) and CL signal obtained in MCR-R neutralization assay (CL signals are background-corrected, dotted line shows threshold value for positive samples (10 AU mL^{-1}), 800 AU mL^{-1} represents upper limit of quantification of YHLO assay, gray line shows linear fit of data).

References

1. Kunze A, Dilcher M, Abd El Wahed A, Hufert F, Niessner R, Seidel M. On-Chip Isothermal Nucleic Acid Amplification on Flow-Based Chemiluminescence Microarray Analysis Platform for the Detection of Viruses and Bacteria. Anal Chem. 2016; https://doi.org/10.1021/acs.analchem.5b03540
2. Seidel M, Niessner R. Chemiluminescence microarrays in analytical chemistry: a critical review. Anal Bioanal Chem. 2014; https://doi.org/10.1007/s00216-014-7968-4
3. Wolter A, Niessner R, Seidel M. Preparation and characterization of functional poly(ethylene glycol) surfaces for the use of antibody microarrays. Anal Chem. 2007; https://doi.org/10.1021/ac070243a
4. Kober C, Niessner R, Seidel M. Quantification of viable and non-viable Legionella spp. by heterogeneous asymmetric recombinase polymerase amplification (haRPA) on a flow-based chemiluminescence microarray. Biosens Bioelectron. 2018; https://doi.org/10.1016/j.bios.2017.08.053
5. Wutz K, Meyer VK, Wacheck S, Krol P, Gareis M, Nölting C, Struck F, Soutschek E, Böcher O, Niessner R, Seidel M. New route for fast detection of antibodies against zoonotic pathogens in sera of slaughtered pigs by means of flow-through chemiluminescence immunochips. Anal Chem. 2013; https://doi.org/10.1021/ac400781t
6. Klüpfel J, Paßreiter S, Weidlein N, Knopp M, Ungerer M, Protzer U, Knolle P, Hayden O, Elsner M, Seidel M. Fully Automated Chemiluminescence Microarray Analysis Platform for Rapid and Multiplexed SARS-CoV-2 Serodiagnostics. Anal Chem. 2022; https://doi.org/10.1021/acs.analchem.1c04672
7. Wu L, Zhou L, Mo M, Liu T, Wu C, Gong C, Lu K, Gong L, Zhu W, Xu Z. SARS-CoV-2 Omicron RBD shows weaker binding affinity than the currently dominant Delta variant to human ACE2. Signal Transduct Target Ther. 2022; https://doi.org/10.1038/s41392-021-00863-2
8. Wang Y, Liu C, Zhang C, Wang Y, Hong Q, Xu S, Li Z, Yang Y, Huang Z, Cong Y. Structural basis for SARS-CoV-2 Delta variant recognition of ACE2 receptor and broadly neutralizing antibodies. Nat Commun. 2022; https://doi.org/10.1038/s41467-022-28528-w
